# Flexible Actuators Based on Conductive Polymer Ionogels and Their Electromechanical Modeling

**DOI:** 10.3390/polym15234482

**Published:** 2023-11-22

**Authors:** Jiawei Xu, Hongwei Hu, Shengtao Zhang, Guanggui Cheng, Jianning Ding

**Affiliations:** 1 School of Mechanical Engineering, Jiangsu University, Zhenjiang 212000, China; 2 Technological Institute of Carbon Neutralization, School of Mechanical Engineering, Yangzhou University, Yangzhou 225000, China

**Keywords:** flexible actuator, electromechanical model, conductive polymer, drive travel, load capacity

## Abstract

High-performance flexible actuators, integral components of soft robotics, hold promise for advancing applications in safe human–robot interactions, healthcare, and various other fields. Notable among these actuators are flexible electrochemical systems, recognized for their merits in low-voltage manipulation, rapid response speed, and cost-effectiveness. However, the optimization of output strain, response speed, and stability presents a significant challenge in this domain. Despite the application of diverse electrochemically active materials to enhance actuation performance, a critical need persists for corresponding electrical-mechanical models to comprehensively grasp actuation mechanisms. In this study, we introduce a novel electrochemical actuator that utilizes conductive polymer ionogel as active electrodes. This ionogel exhibits exceptional properties, including high conductivity, flexibility, and electrochemical activity. Our electrochemical actuators exhibit noteworthy bending strain capabilities and rapid response rates, achieving frequencies up to 10 Hz at a modest voltage of 1 V. An analytical model integrating ion migration and dynamic processes has been established to elucidate actuator behavior. Simulation results highlight that electrodes characterized by low resistance and high capacitance are optimal for simultaneous enhancement of bending strain and blocking force. However, the augmentation of Young’s modulus, while increasing blocking force, compromises bending strain. Furthermore, a larger aspect ratio proves beneficial for unidirectional stress output, leading to increased bending strain, while actuator blocking force diminishes with greater length. These findings underscore the intricate interplay between material properties and dimensions in optimizing the performance of flexible electrochemical actuators. This work provides important practical and theoretical guidance for the manufacture of high-performance flexible actuators and the search for new smart materials.

## 1. Introduction

Biology has long been an inspiration for engineers to build more capable machines. By emulating the adaptability and agility of soft-bodied animals and muscles, soft actuators have emerged with diverse applications encompassing soft grippers, artificial muscles, and smart medical rehabilitation devices [1,2,3]. In this domain, electrochemical actuators assert themselves as formidable contenders due to their inherent conformable and elastic nature, cost-effectiveness, low operational voltage, and air-working capabilities [4]. Notably, extensive research has focused on ionic polymer-metal composites (IPMCs) over the past few decades; however, their practicality was hindered by the reliance on aqueous electrolytes [5,6]. Fukushima et al. introduced a dry-type bucky-gel actuator that utilizes carbon nanotubes (CNTs) as electrodes and embeds an ionic liquid within a polymer matrix as the electrolyte [7,8]. Ionic liquid-based actuators offer the advantage of operating in air with prolonged stability, attributed to the non-volatile nature of ionic liquids. Subsequent research has delved into the utilization of various materials, such as graphene, graphdiyne, and two-dimensional nanomaterials, as active electrodes for ionic liquid-based airworking electrochemical actuators [9,10,11]. Notably, these materials demonstrate elevated electrochemical activities, leading to a substantial enhancement in actuation performance. However, the utilization of these electrochemically active electrode materials typically involves the compounding of these materials with polymers to achieve stable and flexible electrodes, which adds complexity to the manufacturing process, with potential implications for factors like production efficiency and cost. The pursuit of exceptional figures of merit, encompassing actuation strain, response speed, and output force, remains an ongoing challenge.

Conductive polymers (CPs) have earned significant utilization as electrodes for electrochemical energy storage, owing to their heightened electrochemical activity and electronic conductivity—two pivotal attributes for enabling robust electrochemical actuation [12,13]. Ionogels have garnered significant interest in the realm of flexible electronics by combining solid-state networks with ionic liquids. This innovative approach not only offers outstanding mechanical properties and conductivity to address the challenges in flexible electronics but also brings another distinctive feature: high electrochemical activity for electrochemical actuators. Previously, bucky-gel type ionogels, comprising carbon nanotubes (CNT) and ionic liquids, have demonstrated remarkable electrochemical actuation capabilities. The incorporation of conductive fillers in ionogels has proven effective in elevating electrochemical activities and augmenting actuation performance. Consequently, the exploration of conductive polymer-based ionogels for electrochemical actuation presents a promising avenue for advancing the current capabilities of conductive polymer-based electrochemical actuators.

In this study, we introduce CP ionogels characterized by hierarchical layered structures that integrate ionic liquids into polymer matrixes. These ionogels exhibit superior electrical conductivity (500 S/cm) and electrochemical activity. Electrochemical actuators built upon CP ionogels demonstrate remarkable strain capabilities (1.2%) and rapid response rates (up to 10 Hz) while operating at a modest voltage of 1 V. Furthermore, we have established an electromechanical model for the ionogel actuator, closely aligning the electrochemical response with mechanical properties flexible actuators. Leveraging this model, we systematically scrutinized crucial parameters influencing actuator performance, encompassing electrode conductivity, Young’s modulus, capacitive characteristics, and actuator dimensions. These results indicate that the optimal characteristics of electrodes featuring low resistance and high capacitance facilitate a concurrent improvement in bending strain and blocking force. However, the elevation of Young’s modulus, while bolstering blocking force, comes at the expense of bending strain. Additionally, a larger aspect ratio proves advantageous for unidirectional stress output, leading to amplified bending strain, whereas actuator blocking force undergoes a reduction with increased length. This comprehensive investigation offers insight into the intricate interplay among these physical factors and their influence on actuator displacement and blocking force. This research forms an essential foundation, offering significant guidance for the ensuing design and optimization of novel ionogel-based actuators.

## 2. Experimental and Simulation Method

### 2.1. Design and Operating Principle

The fabricated electrochemical actuators consist of three layers: two electroactive layers (conductive polymer ionogel films), which serve as both electrodes and actuation layers, with an electrolyte gel layer in between. When the two electrodes are connected to drive voltages, cations, and anions migrate within the electrolyte and ion gel electrodes due to internal electric fields, leading to the swelling of one conductive polymer ionogel and the contraction of the other. Different deformations of the two electroactive layers produce strain mismatch, which has been studied in our previous work [14,15]. This strain mismatch mechanism causes the actuator to convert strain energy into deformation, resulting in the trilayer actuator bending in one direction.

### 2.2. Materials

Ionic liquids including EMIMTFSI (1-Ethyl-3-Methylimidazolium Bis(Trifluorome- thylsulfonyl)Imide and BMImOTs 1-butyl-3-methylimidazolium tosylate were purchased from Greenchem ILs, LICP, CAS, China (Lanzhou, China); PEDOT:PSS aqueous solution (solid content 1.0–1.3 wt%, PEDOT to PSS ratio is 1:2.5, OE800) was purchased from Shanghai Ouyi Organic Optoelectronic Materials Co., LTD., Shanghia, China

### 2.3. Fabrication of Flexible Actuator Based on the CP Ionogel

CP film was fabricated by casting PEDOT:PSS dispersion, drying, and annealing. CP ionogel was then formed by treating the CP film in an ionic liquid. Specifically, ionic additives (BMImOTs, 15 wt%) were added to the PEDOT:PSS solution. After stirring for 1 h and sonication for 15 min, the solution was poured into a Teflon mold and left to stand at room temperature for 24 h. It was then transferred to an electric oven treated at 60 °C for 2 h and 150 °C for 30 min. After cooling to room temperature, the film was peeled off and subsequently immersed in ethanol and DI water. The ionogel was prepared by immersing the swelled film in ionic liquid (EMIMTFSI) and placed at 80 °C for 4 h. The film was then removed and dried in a vacuum oven overnight. Two ionogel electrode films were laminated on a cellulose membrane film that was pre-soaked by ionic liquid (EMIMTFSI). They were then pressed together using two glass slides and placed at 60 °C for 20 min. Afterward, the trilayer was carefully peeled off from the glass slides. All the actuators were cut into long strips with 5 mm width and 26 mm length.

### 2.4. Characterization and Measurement

The electronic conductivity of the films was tested using a four-point probe station (HPS2523, HELPASS Electronic Technologies Ltd., Changzhou, China). The strain-stress curve was performed on a mechanical tester (QT-6203S, Qiantong Instrument Equipment Co., Ltd., Suzhou, China). Cross-sectional SEM images were shot on a JEOL 7800F (JEOL Ltd., Akishima, Japan). The performance of the actuators was measured using a dual channel source meter (Keithley 2602B, Tektronix Inc., West Chester, OH, USA) for the input power and a laser displacement meter to record the displacement. One end of the actuator was fixed by a Kelvin clamp with two platinum plates on the contact area, and the swing of the other end was recorded by the laser displacement meter. The strain (ε) of the actuator can be calculated by the following equation:

$$\delta =\frac{2d\theta }{L^{2}+\delta^{2}}$$

where δ,d, and *L* are the tip displacement, the thickness, and the beam length, respectively. The blocking force was measured using a high-precision pressure transducer (Futek LSB200, FUTEK Advanced Sensor Technology, Inc., Irvine, CA, USA) with the probe attached horizontally to the tip of the actuator beam.

### 2.5. Simulation Method

To further analyze the mechanical behavior of the CP ionogel actuator and identify the key parameters affecting the actuation performance, it is necessary to establish an analytical model containing the necessary physical quantities [16]. We use a Multistage parallel equivalent circuit to simplify the electrical part of the actuator. A single-ended fixed beam model simplifies the statics part, and a pole-zero model is used to couple the electrical and mechanical parts. The parameters of the Pole-zero model are identified using experimental data. Finally, a gray-box model about the control voltage u and the displacement of end position δ with certain physical parameters that can reflect the electrical and mechanical properties of the system is obtained. To establish the analytical model of the actuator, some appropriate assumptions are made as follows:

1. Since the surface resistance of electrodes is very small ($R_{s}\approx$ 14 Ω), it is assumed that the resistances in each stage of the RC circuit are equal: $R_{n}=R_{s}/n$.
2. Since the thickness of the capacitor layer is very small (0.14 mm), the micro-element capacity of the capacitor in the radial direction is equal.
3. The bending of the actuator is uniform along the whole beam.
4. The influence of bending on the electrical properties of the capacitor layer is negligible.
5. The driving force comes from the ionogel electrode layer with a uniform stress distribution.

## 3. Results and Discussion

### 3.1. Driving Characteristics of the Flexible Actuator

**Figure 1:**
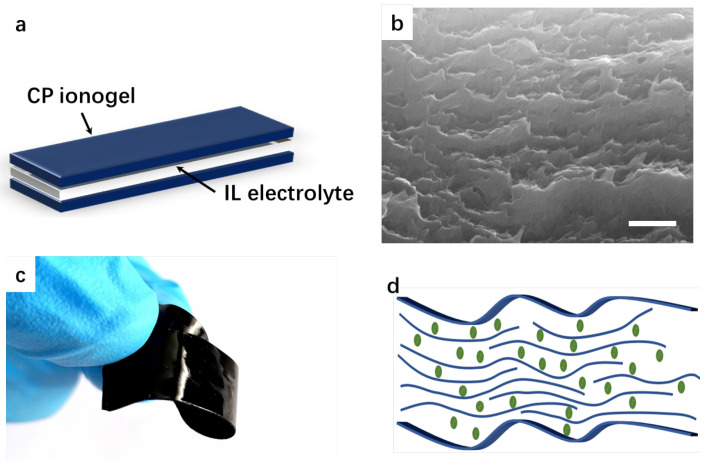
(**a**) structure of the soft actuator based on CP ionogel; (**b**) SEM image of the microstructure of CP film (scale bar: 500 nm); (**c**) photo of the CP ionogel; (**d**) illustration of the microstructure of the CP ionogel, where green dots represent the ionic liquid.

The flexible actuator exhibits a sandwich structure consisting of two conducting polymer ion gel electrodes and a quasi-solid ionic liquid electrolyte membrane in the middle (Figure 1a). CP ionogel membranes were prepared from PEDOT:PSS aqueous dispersions with ionic additives to improve membrane quality, including electrical conductivity, electrochemical activity, and mechanical properties, which are all important for driving performance. The introduction of ionic additives into the PEDOT:PSS dispersion can reduce the electrostatic force between PEDOT and PSS, thereby rearranging the molecular chains, thereby improving the interchain interactions during solvent evaporation and recrystallization, forming a molecular chain network structure [17]. After annealing, the PEDOT:PSS film has a layered nano-stacked structure, as shown in the SEM image of Figure 1b, which also facilitates the intercalation of ionic liquids. When immersed in ionic liquids, the films with layered structures can absorb 15 wt% of ionic liquids, forming soft conducting polymer ionogel (CP ionogel, Figure 1c,d).

**Figure 2:**
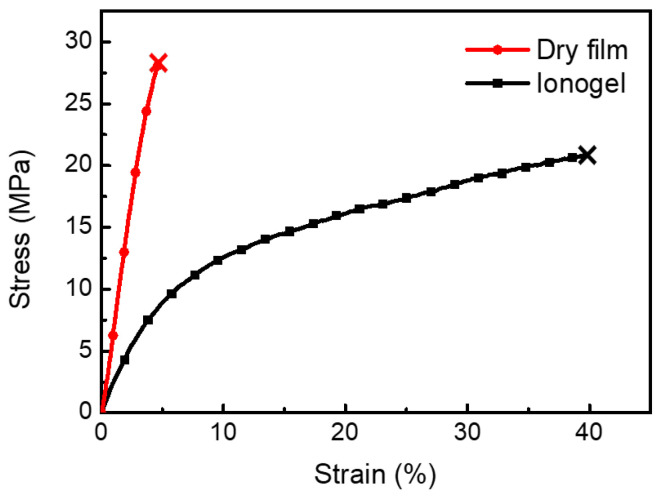
Tensile stress–strain curve of the CP dry and ionogel films.

Ionic liquids in CP ionogels not only effectively reduce Young’s modulus of PEDOT:PSS film and render it stretchable but also increase the conductivity. In tensile tests (Figure 2), the dry CP film breaks at 4.6% strain with a high Young’s modulus of 700 MPa, while the CP ionogel can be stretched up to 40% with a tensile strength of 20.8 MPa and a low Young’s modulus of 217 MPa, which is due to the plasticizing effect of the ionic liquid. Furthermore, the electrical conductivity of the conducting polymer ionogel can reach 500 S/cm, compared with 20 S/cm for dry CP film. The enhanced conductivity can be attributed to the doping effect from the presence of ionic liquid in the ionogels [17].

**Figure 3:**
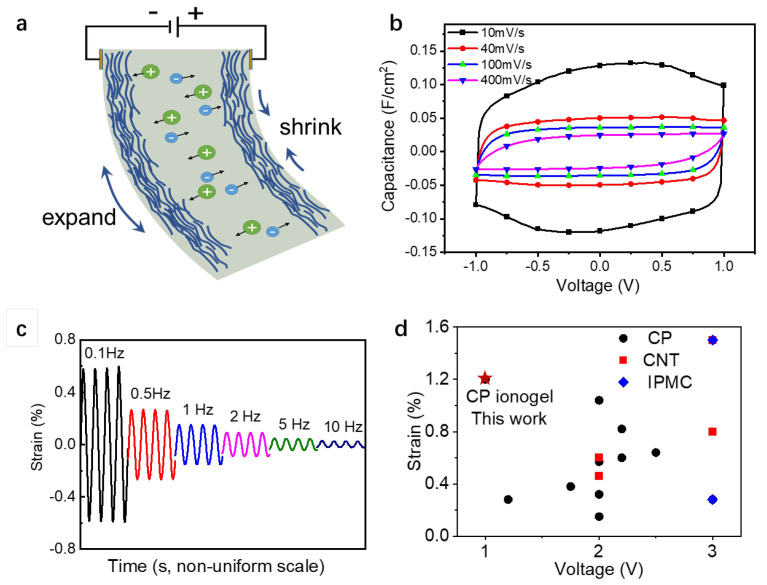
Actuation performance of the CP ionogel actuator. (**a**) illustration of the actuation mechanism of CP ionogel actuator; (**b**) CV curves of the actuator at different scan rates; (**c**) strain of the actuator under different frequencies of triangular voltage between ±1 V; (**d**) comparison of the strain with reported works [19,20,21,22,23,24,25,26,27,28].

The soft actuator, consisting of two CP ionogels and a quasi-solid electrolyte membrane, acts like a supercapacitor, storing charge during operation. Previous studies have shown that CP films with polymeric counterions, such as PSS, are mainly driven by cationic motion [18]. Therefore, the introduction of cations on the negative side causes the film to swell, while the removal of cations on the positive side causes the film to shrink (Figure 3a). The cyclic voltammetric characters of the actuator were examined at different scan rates (Figure 3b). At a slow sweep speed of 10 mV/s, the CV curve is approximately rectangular, indicating that the actuator has a double-layer charge storage characteristic similar to that of a supercapacitor. As the sweep speed increases, the capacitance decreases, and the CV curve gradually becomes conical at the fast sweep speed, indicating that the ion migration rate is the main reason for the capacitance decrease at the fast sweep speed.

We tested the actuators operated at varied frequencies from 0.1 Hz to 10 Hz with a fixed amplitude of 1 V (Figure 3c). The maximum strain difference at 0.1 Hz reaches 1.2%. By increasing the applied voltage frequency, the maximum strain decreased, revealing that the slow kinetics of the electrochemical process limited the actuation at a higher frequency. It is worth noting that the strain of CP ionogel actuators is among the highest values from reported works on CP-based actuators (Figure 3d). Furthermore, by comparing with other types of actuators, we found that CP ionogel actuators can provide strain values that other actuators can only achieve at higher driving voltages, which suggests that CP ionogel actuators are promising for flexible wearable devices requiring low energy consumption.

### 3.2. Modeling

**Figure 4:**
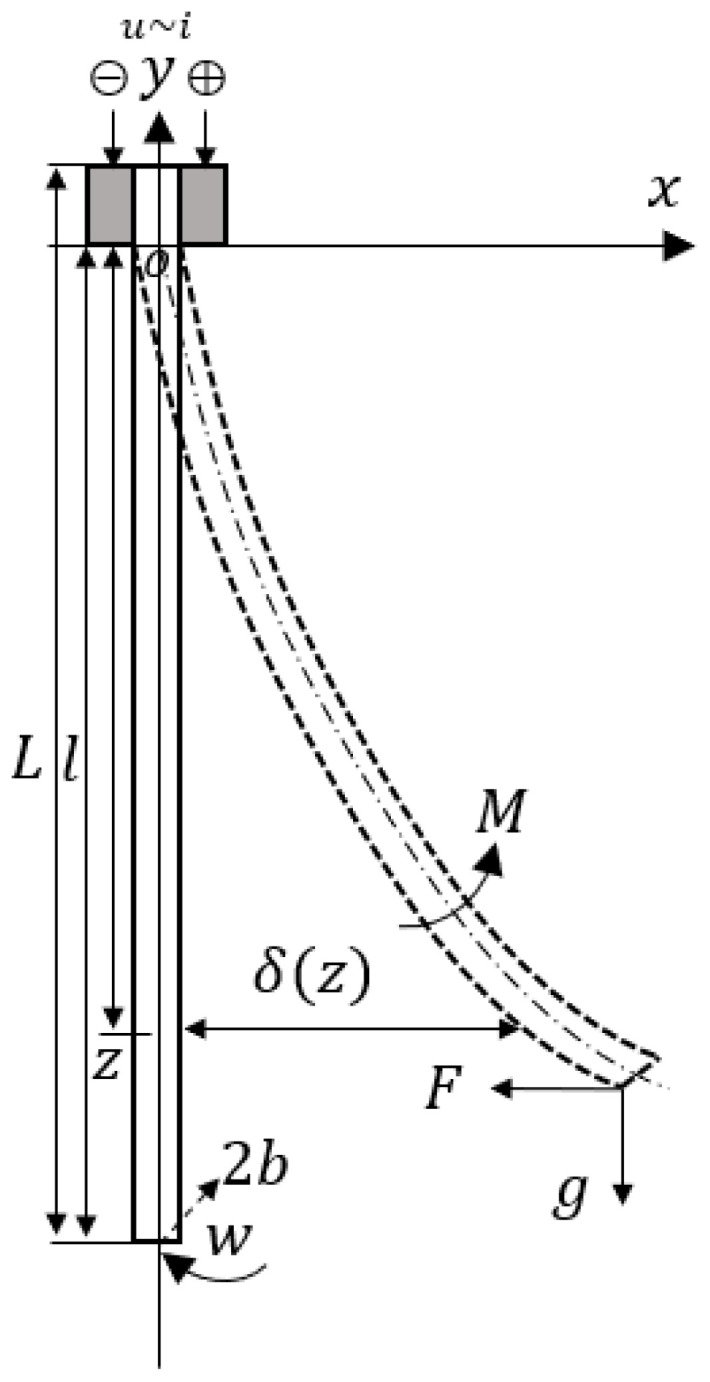
Physical parameters of the actuator for modeling.

The physical structure diagram of the ionic actuator is shown in Figure 4. A section of the actuator is clamped by gold electrodes and keeps the whole actuator hanging freely. The specific meaning of each physical quantity is detailed in Table 1.

**Figure 5:**
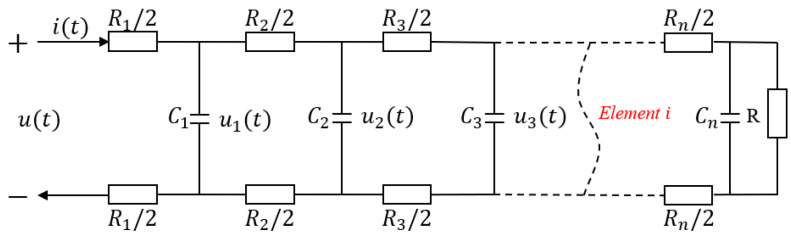
The equivalent electrical circuit model for the actuator.

In the electrical part, the electrical model of the actuator is established by simulating the actuator using a simplified multi-level equivalent RC circuit [29] (Figure 5), where Rn/2 is the sheet resistance of each unit in the single-layer ionogel, and Ci is the capacitance of each unit in the electrolyte layer. Through the physical meaning of resistance and capacitance, we can easily obtain

(1) $$R_{i}=\frac{2\rho_{i}b}{Lw},C_{i}=\frac{\varepsilon_{i}Lw}{2b}$$

where $\rho_{i}$ represents the resistivity of each micro-element circuit resistance, and $\varepsilon_{i}$ represents the dielectric constant of the capacitance of each micro-element circuit. The complete state space model of the equivalent circuit is given as follows:

(2) $$\begin{array}{c} \left\{\begin{array}{cc} C_{1}\dot{u_{1}}\left(t\right) & =\frac{u\left(t\right)-u_{1}\left(t\right)}{R_{1}}-\frac{u_{1}\left(t\right)-u_{2}\left(t\right)}{R_{2}}\\ C_{2}{\dot{u}}_{2}\left(t\right) & =\frac{u_{1}\left(t\right)-u_{2}\left(t\right)}{R_{2}}-\frac{u_{2}\left(t\right)-u_{3}\left(t\right)}{R_{3}}\\ \; & \;\vdots \\ C_{n-1}{\dot{u}}_{n-1} & =\frac{u_{n-2}\left(t\right)-u_{n-1}\left(t\right)}{R_{n-1}}-\frac{u_{n-1}\left(t\right)-u_{n}\left(t\right)}{R_{n}}\\ C_{n}{\dot{u}}_{n}\left(t\right) & =\frac{u_{n-1}-u_{n}\left(t\right)}{R_{n}}-\frac{u_{n}\left(t\right)}{R} \end{array}\right. \end{array}$$

where $u_{n}$ is the voltage on each RC shunt, this state space model is used to describe the charge transfer process in the channels, all the mathematical derivation in this paper take place in Maple (2022 version).

The number of impedance elements will determine the available degrees of freedom to describe the dynamics of the electrical model [30]. In general, as the number of impedance elements increases, the accuracy of the model will increase. However, this will result in increasingly complex calculations. When the ladder circuit operates at low frequencies, it can be reduced to a suitably low order to achieve a given accuracy [31]. To balance the accuracy and complexity, the second-order link is used as an example for calculation. The transfer function between its charge Q and the input voltage u can be written as follows, where the variable ‘s’ represents the differential operator in the s-domain:

(3) $$G_{Qu}\left(s\right)=\frac{RR_{1}C_{1}C_{2}s^{2}+\left(RC_{1}+R_{2}C_{1}+RC_{2}\right)s}{RR_{1}R_{2}C_{1}C_{2}s^{2}+\left(R_{1}R_{2}C_{1}+RR_{1}C_{1}+RR_{1}C_{2}+RR_{2}C_{2}\right)s+\left(R_{1}+R_{2}+R\right)}$$

**Figure 6:**
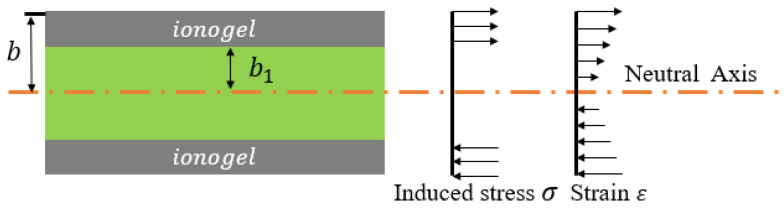
The mechanical model of the actuator.

The migration of ions driven by the electric field causes the electrode on one side of the actuator to expand and the electrode on the other side to contract. Obviously, if the stress due to volume expansion is assumed to be equal to the stress due to volume reduction, then the entire multilayer model can be simplified to a single layer for the mechanical analysis. Therefore, based on the mechanical model shown in Figure 6, the transfer function of the moment M(s) generated by the induced stress can be written as

(4) $$\begin{array}{cc} M(s) & =2\int_{b1}^{b}\sigma_{c}\left(s\right)ywdy,\\ \; & =w\sigma_{c}\left(s\right)\left(b^{2}-b_{1}^{2}\right), \end{array}$$

where $\sigma_{c}\left(s\right)$ is the induced stress caused by the volume change, and w,b, and $b_{1}$ are the width of the membrane, half of the overall thickness of the actuator, and the thickness of the electrode layer, respectively. The induced stress arises from the transformation of the volume, so the volume stress is used here to calculate the deformation due to charge transfer. The electromechanical coupler is introduced to couple the change of charge amount with the induced stress. Here, we choose the pole-zero model, which is shown in function (6).

(5) $$\sigma_{c}\left(s\right)=\frac{Q(s)d(s)}{V}$$

where $V=Lw(b-b_{1})$ and *L* is the overall length of the actuator. d(s) is the electromechanical coupling equation.

(6) $$d\left(s\right)=\frac{k\prod_{j=1}^{m}\left(s+z_{j}\right)}{\prod_{i=1}^{n}\left(s+p_{i}\right)}$$

where $k,m,n,z_{j},$ and $p_{i}$ are the parameters to be tuned. Here, k is the gain of the system, m and n are the number of poles and zeros, and $z_{j}$, and $p_{i}$ are the positions of the zeros and poles to be tuned in the frequency domain. Substituting Equation (5) into Equation (4), we obtain

(7) $$M\left(s\right)=\frac{\left(b+b_{1}\right)Q\left(s\right)d\left(s\right)}{L}$$

Through the mechanical balance of the actuator, it is not difficult to obtain the following relationship:

(8) $$\sum_{i}=M\left(s\right)-\left(F+g\right)l-\int_{b}^{-b}\sigma_{g}\left(s,x\right)wxdx=0$$

where $\sigma_{g}\left(s,x\right)$ is the internal stress existing against elastic deformation and (F+g)l is the equivalent concentrated stress on the end of the actuator and the moment generated by gravity. The internal stress can be written as:

(9) $$\sigma_{g}\left(s,x\right)=E\kappa \left(s\right)x$$

where E is Young’s modulus, and κ is the bending curvature of the actuator. Since the weight of the actuator is very light, and there is no blocking force at the end, we set (F+g)l=0 at this time to simplify the calculation. Therefore, we obtain

(10) $$\begin{array}{cc} M(s) & =\int_{b}^{-b}\sigma_{g}\left(s,x\right)wxdx\\ \; & =\frac{2}{3}Ewb^{3}\kappa \left(s\right) \end{array}$$

Putting Equation (7) into Equation (10), we obtain

(11) $$\kappa \left(s\right)=\frac{3\left(b+b_{1}\right)Q\left(s\right)d\left(s\right)}{2Ewlb^{3}}$$

Therefore, the axial displacement can be approximated as follows:

(12) $$\delta \left(z,s\right)=\frac{1-cos(z\kappa (s))}{\kappa (s)}$$

where *z* is the observed inflection point.

Here we approximate δ(z,s) by Taylor series expansions of κ,z at the zero point:

(13) $$\delta \left(z,s\right)\approx \frac{1}{2}z^{2}\kappa \left(s\right)$$

Finally, combining Equations (3), (11) and (13), the transfer function $G_{\delta u}$ of the input voltage u(s) to the bending displacement output δ(z,s) can be written as:

(14) $$G_{\delta u}=\frac{\delta (z,s)}{u(s)}=\frac{3z^{2}\left(b+b_{1}\right)G_{Qu}\left(s\right)d\left(s\right)}{4Lwb^{3}E}$$

With a simple deformation of Equation (8), it is not difficult to obtain the relationship between the blocking force F and the driving voltage *u*:

(15) $$G_{Fu}=\frac{\left(b+b_{1}\right)G_{Qu}d\left(s\right)}{15L}$$

Then, the modeling of the CP ionogel actuator is completed.

### 3.3. Parameter Identification and Verification

In this section, we demonstrated an actuator for parameter identification. The total length is 26 mm, the width is 5 mm, and the average thickness is 0.18 mm. The free length of the actuator is 20 mm. We use a step signal with a voltage of 1 V for system parameter identification. Based on the assumptions in the previous section, only the parameters of the electromechanical coupling equations need to be identified. The parameters used in the model from experiment data are shown in Table 2. Here, we use the least squares method for parameter identification, and the cost function is as follows:

(16) $$min\sum {\left(f\left(x_{i}\right)-y_{i}\right)}^{2}$$

where $f(x_{i})$ is the system function to be identified, and *y* is the experimental value.

Here, two zeros and two poles are used as the basic parameters of the electromechanical coupling model, and the parameters to be identified are $k,z_{1},z_{2},p_{1}$, and $p_{2}$. By performing least squares fitting in MATLAB, all the system parameters are obtained, which are shown in Table 3.

**Figure 7:**
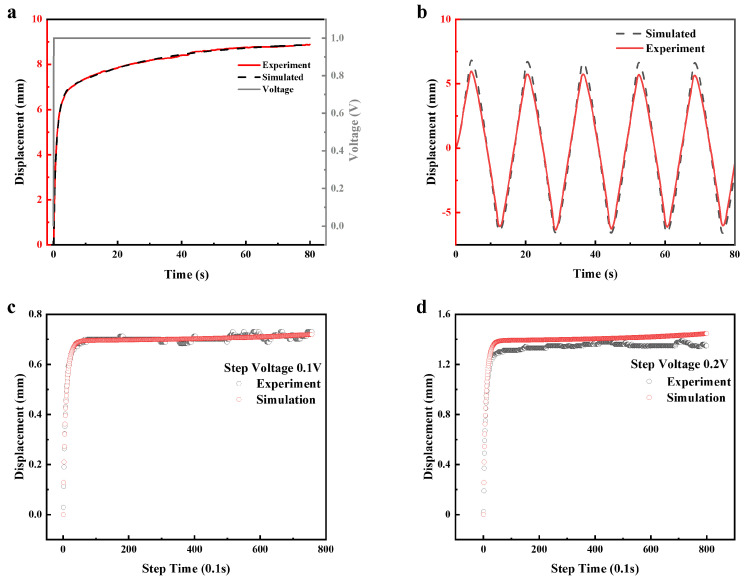
Verification of the model by comparing the experimental and simulated results ((**a**) 1 V step voltage; (**b**) 1 V ramping voltage; (**c**) 0.1 V step voltage; (**d**) 0.2 V step voltage). Difference between simulation results based on analytical model and experimental data. (The structural parameters and electrical parameters of the experiment are consistent with those of the simulation).

To verify the accuracy of our analytical model, we tested the displacement response of a 20 mm long actuator (total length 26 mm, the electrode clamping part length is 6 mm) at a 1 V step voltage. The experimental data and the theoretical data derived from the model show very high agreement (Figure 7a). Furthermore, when the actuator works at a ramping voltage of 1 V, its displacement response is in good agreement with the simulation results. The deviation range of its maximum displacement is within 10% (Figure 7b). The presence of very small variations in film properties or internal stresses during actuator preparation can introduce disparities in the strain values when the actuator bends in both directions under alternating voltage. At the same time, in the experimental test, the different stiffness of the conductive clamp spring will produce different holding forces, which will also lead to the accumulation of errors in the test. Furthermore, we test the prediction performance of the model under different step voltages (0.1 V and 0.2 V), which are not covered in fitting (Figure 7c,d); the results show that the predicted and experimental data are generally moderate, although the test results showed some deviation when excited by a 0.2 V step voltage, as mentioned earlier, the accumulation of errors during the experimental process can lead to an offset in the test results. However, as shown in the figure, the maximum deviation between the experimental and predicted values is less than 0.2 mm. These results show that our model can well reflect the dynamic response of the actuator and thus make a good prediction of the displacement of the actuator’s end position.

### 3.4. Analysis Result

**Figure 8:**
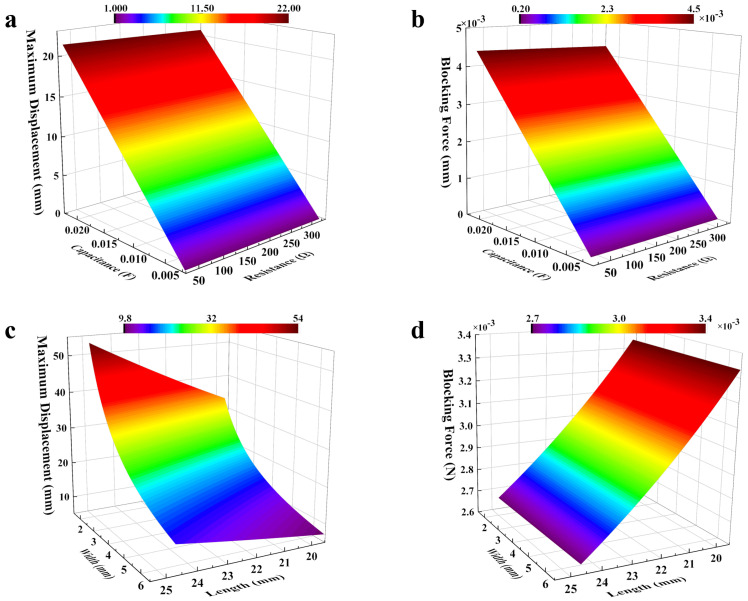
(**a**,**b**) The influence of electrode resistance and capacitance on maximum displacement at the end of the actuator and blocking force; (**c**,**d**) The relationship of actuator’s length and width between displacement at the end of the actuator and blocking force.

Based on the established electromechanical coupling model, we further discuss the influence of various parameters of the actuator on the actuation performance. Because the ion migration velocity is reflected in capacitor size, in this section, the concept of equivalent capacitance is introduced. Figure 8a,b shows the end position displacement and blocking force of the actuator as a function of the electrode resistance and capacitance. When the resistance is increased from 10 Ω to 300 Ω, the displacement decreases from 21.9 mm to 21.3 mm, while the blocking force drops from 4.47 mN to 4.35 mN. On the other hand, when the capacitance increases from 0.001 F to 0.02 F, the displacement increases from 1.1 mm to 21.9 mm, while the blocking force improves from 0.2 mN to 4.47 mN. These results show that the capacitance of the electrodes has a large effect on both displacement and blocking force. In contrast, as long as the resistance is within a few hundred orders of magnitude, the resistance has little effect on the actuator. Based on the simulation results, electrodes with low resistive and high capacitive properties can simultaneously generate greater displacement and blocking force, making them ideal candidates for actuator materials.

**Figure 9:**
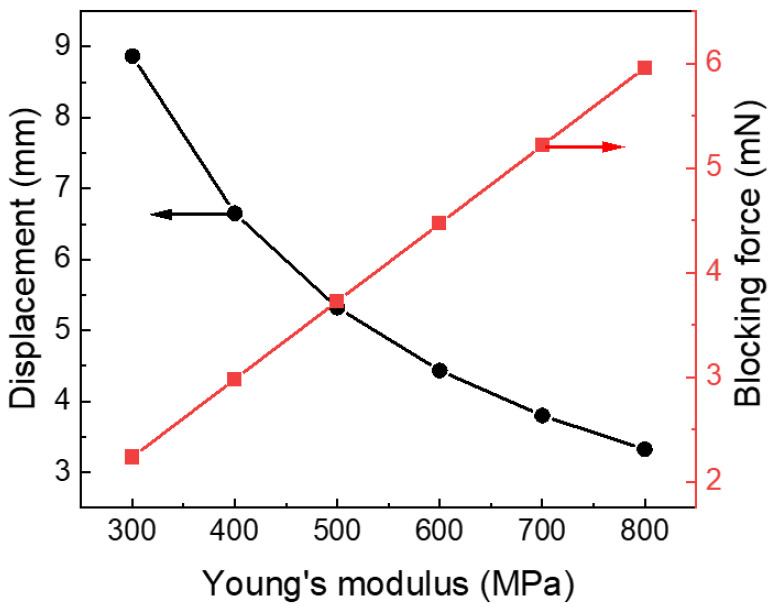
The displacement of end position and blocking force of the actuator based on its Young’s Modulus.

The mechanical properties of the electrodes also have a very important influence on the performance of the actuator. It can be seen from Figure 9 that although the improvement of Young’s modulus can improve the blocking force of the actuator, it also leads to a decrease in displacement. Therefore, it is necessary to select electrode materials with appropriate mechanical properties based on comprehensive consideration of the requirements for strain and gripping force in practical applications.

Optimizing the size of the actuator can also increase the output of its performance. By increasing the length of the actuator, the tip displacement is gradually increased (Figure 8c,d). The displacement of the end of the actuator can be further increased by using a narrower beam. Thus, a larger aspect ratio is beneficial for the unidirectional output of internal stress, resulting in a larger displacement. The blocking force of the actuator decreases with increasing length, while width has a trivial effect on it. This is because the moment generated by the system is only related to the voltage. When the electrical parameters are fixed, the moment generated by the system is also fixed, so a longer length means a longer arm of force, which reduces the blocking force.

Hence, to obtain a high load, large deformation, and fast response, the actuator needs higher capacitance, and Young’s modulus should be controlled at around 500 MPa. For the structure of the actuator, reducing the length and increasing the width can achieve greater load capacity, and vice versa can obtain greater displacement of the end position.

## 4. Conclusions

In this work, a novel electrochemical actuator with large strain and low operating voltage was developed using CP ionogel as the electrode. Due to the high electrical conductivity, flexibility, and excellent electrochemical activity of ionogels, the flexible actuator in this study outperforms most others employing materials like graphene and graphdiyne. Additionally, the preparation process is considerably simpler. An analytical model of the actuator by combining the equivalent electrical and motion processes is established, with the largest disagreement between simulated and the corresponding measured data being less than 10%. In addition, our study systematically scrutinized key parameters influencing actuator performance, including electrode resistance, capacitance, Young’s modulus, and actuator size. Simulation results underscore the optimal nature of electrodes with low resistance and high capacitance, contributing to the simultaneous enhancement of bending strain and blocking force. Nevertheless, the increase of Young’s modulus, despite augmenting blocking force, compromises bending strain. Moreover, a larger aspect ratio emerges as advantageous for unidirectional stress output, resulting in increased bending strain, while actuator blocking force experiences a reduction with greater length. This thorough investigation elucidates the intricate interplay of these physical factors and their impact on actuator displacement and blocking force. Our work provides valuable practical insights and theoretical guidance for the future development of high-performance electrochemical actuators and the exploration of innovative smart materials.

## Figures and Tables

**Table 1:** Model Parameters.

Parameters	Meanings
L	Total Length
*l*	Free Length
*z*	Observation Point
F	Blocking Force
δ(*z*)	Displacement of Observation point
*w*	Width
*b*	Half Thickness
*u*	Control Voltage
*i*	Current
*M*	Induced Bending Moment caused by Ionic Migration
*g*	Gravity

**Table 2:** Parameters of Modeling.

Parameters	Values
$R_{1},R_{2}$	7 Ω
$C_{1},C_{2}$	0.01 F
*R*	10,000 Ω
*L*	20 mm
*E*	300 MPa
*w*	4.2 mm
*z*	18 mm
*b*	0.09 mm
$b_{1}$	0.02 mm

**Table 3:** Parameters of Electromechanical Coupler Function.

Parameters	Values
k	86.478
p1	0.036
p2	0.901
z1	2.406
z2	0.048

## Data Availability

The experimental data on the results reported in this manuscript are available upon an official request to the corresponding author.
